# Exposure to alcohol outlets and risk of suicidal behavior in a Swedish cohort of young adults

**DOI:** 10.1111/acer.15051

**Published:** 2023-05-29

**Authors:** Alexis C. Edwards, Henrik Ohlsson, Séverine Lannoy, Mallory Stephenson, Casey Crump, Jan Sundquist, Kenneth S. Kendler, Kristina Sundquist

**Affiliations:** 1 Virginia Institute for Psychiatric and Behavioral Genetics, Virginia Commonwealth University, Richmond, Virginia, USA; 2 Department of Psychiatry, Virginia Commonwealth University, Richmond, Virginia, USA; 3 Center for Primary Health Care Research, Lund University, Malmö, Sweden; 4 Integrative Life Sciences, Virginia Commonwealth University, Richmond, Virginia, USA; 5 Department of Population Health Science and Policy, Icahn School of Medicine at Mount Sinai, New York, New York, USA

**Keywords:** alcohol outlets, gene-environment interaction, suicidal behavior, suicide attempt

## Abstract

**Background:**

Greater alcohol accessibility, for example in the form of a high density of alcohol outlets or low alcohol taxation rates, may be associated with increased risk of suicidal behavior. However, most studies have been conducted at the aggregate level, and some have not accounted for potential confounders such as socioeconomic position or neighborhood quality.

**Methods:**

In a Swedish cohort of young adults aged 18 to 25, we used logistic regressions to evaluate whether living in a neighborhood that included bars, nightclubs, and/or government alcohol outlets was associated with risk of suicide attempt (SA) or suicide death (SD) during four separate 2-year observation periods. Neighborhoods were defined using pre-established nationwide designations. We conducted combined-sex and sex-stratified analyses, and included as covariates indicators of socioeconomic position, neighborhood deprivation, and aggregate genetic liability to suicidal behavior.

**Results:**

Risk of SA was increased in some subsamples of individuals living in a neighborhood with a bar or government alcohol outlet (odds ratios [ORs] = 1.05 to 1.15). Risk of SD was also higher among certain subsamples living in a neighborhood with a government outlet (ORs = 1.47 to 1.56), but lower for those living near a bar (ORs = 0.89 to 0.91). Significant results were driven by, but not exclusive to, the male subsample. Individuals with higher aggregate genetic risk for SA were more sensitive to the effects of a neighborhood government alcohol outlet, pooled across observation periods, in analyses of the sexes combined (relative excess risk due to interaction [RERI] = 0.05; 95% confidence intervals [CI] 0.01; 0.09) and in the male subsample (RERI = 0.06; 95% CI 0.001; 0.12).

**Conclusions:**

Although effect sizes are small, living in a neighborhood with bars and/or government alcohol outlets may increase suicidal behavior among young adults. Individuals with higher genetic liability for SA are slightly more susceptible to these exposures.

## INTRODUCTION

Suicidal behavior, to include attempts and death by suicide, is frequently accompanied by alcohol use and intoxication (Borges et al., 2017; Conner et al., 2014; Conner & Bagge, 2019; Kaplan et al., 2014; Xuan et al., 2016). Indeed, a recent meta-analysis of studies in the United States, Canada, and Mexico estimated the alcohol-attributable fraction of suicide at 0.21 (Alpert et al., 2022). Both acute alcohol use and alcohol use disorder (AUD), which typically develops through chronic alcohol use, are associated with increased risk of suicidal behavior (Norstrom & Rossow, 2016), potentially via different mechanisms (Conner & Duberstein, 2004). Using Swedish national registry data, we have previously demonstrated that AUD is strongly associated with both suicide attempt (SA) and suicide death (SD), likely due to both shared genetic and environmental liability between these outcomes, and to causal pathways (Edwards et al., 2020, 2022).

The correlation between alcohol intoxication or AUD and suicidal behavior raises the question of whether the accessibility of alcohol might contribute to increased risk of SAs or death (Xuan et al., 2016). For example, environments where alcohol use is promoted could lead to more frequent or intense intoxication and/or to AUD and its attendant negative interpersonal, physiological, and psychological sequelae–all of which are associated with increased risk of suicidality. Alcohol accessibility can be operationalized in various ways, including the extent to which alcohol is taxed at the local or state level, whether a municipality prohibits the sale of alcohol, or individual proximity to or density of alcohol outlets. Previous research in this realm has yielded inconsistent findings (Kolves et al., 2020). For example, studies utilizing aggregate data in New Mexico (Escobedo & Ortiz, 2002) and California (Johnson et al., 2009) both reported that higher off-premise outlet densities (e.g., liquor stores) were associated with increased suicide rates. However, Johnson et al. (2009) also found that bar (on-premise) density was associated with both SA and SD. While other studies have provided further support for higher suicide rates in areas with increased alcohol accessibility (e.g., high outlet density and lower age restrictions), associations have in some cases been limited to only males (Giesbrecht et al., 2015; Markowitz et al., 2003), or to young adults but not adolescents (Markowitz et al., 2003). Finally, a study of 22 US states reported that a composite measure of restrictive alcohol-related policies was associated with lower risk of SD by firearm, but not with SD overall (Coleman et al., 2021).

Alcohol outlets represent immediate, visible, physical access to alcohol. This contrasts with, for example, state or local policies that impact penalties for driving while intoxicated, having a false ID, or raise awareness of the risks of drinking while pregnant, etc., of which alcohol users might not even be aware, and which might differentially impact those who consume alcohol. Thus, proximity of alcohol outlets is a relatively unambiguous indicator of alcohol accessibility. Given prior evidence that alcohol consumption and/or intoxication is higher in areas with more alcohol outlets (Campbell et al., 2009), and multiple pathways via which alcohol intoxication and/or AUD can lead to suicidal behavior–including behavioral disinhibition, depressed mood, and aggression (Kõlves et al., 2022)–alcohol outlets are a promising target for questions regarding how alcohol accessibility is related to suicidal behavior.

In this study, we aim to provide insight into the role of accessibility to alcohol outlets in risk of suicidal behavior in a Swedish cohort of young adults aged 18 to 25 by estimating the association between the presence versus the absence of a neighborhood alcohol outlet and SA or SD. We focus on this age range for two primary reasons: (i) This group has a relatively high rate of suicidal behavior, in particular SA (Hadlaczky, 2022), which has received less attention in prior research on alcohol access and suicidality; and (ii) while all individuals of age 20 and above can obtain alcohol from government outlets (Systembolaget, similar to the Alcoholic Beverage Control stores in the United States), young adulthood is a period during which much alcohol consumption occurs at on-premise sites (e.g., bars, which allow drinking at age 18 and above), affording us superior power to detect potential effects of this exposure class. We restrict our observation period to each of four 2-year windows beginning with the date at which exposure to each alcohol outlet was measured. Our rationale for this restriction is that any risk conferred by proximity to an alcohol outlet is more likely to be relatively time-limited rather than extending to several years beyond the exposure.

These efforts complement many prior studies on the association between alcohol outlets and suicidal behavior through our use of individual-level, rather than aggregate, data on proximity to alcohol outlets and suicide outcomes. Furthermore, we are able to examine both fatal and nonfatal outcomes. Because prior studies have identified sex-specific effects, we will leverage our substantial sample size by conducting sex-stratified analyses. As some previous research has been based on specific outlet types, we will distinguish among bars, nightclubs, and government-controlled facilities. Where main effects of outlets are detected, we will assess whether genetic liability to suicidal behavior interacts with these effects: Abundant evidence provides support for a moderate-to-substantial genetic component to suicidal outcomes (Brent & Melhem, 2008; Docherty et al., 2020; Edwards et al., 2021; Mullins et al., 2022), and more permissive environments may lead to increased risk of adverse outcomes among those with higher genetic liability for psychiatric/substance use outcomes (Assary et al., 2018; Kendler et al., 2014; Young-Wolff et al., 2011). Notably, a prior study found that the heritability of drinking frequency was increased among young adults exposed to a higher density of alcohol outlets (Slutske et al., 2019), but to our knowledge, a comparable analysis focused on genetic liability to suicidal behavior has not been reported. We hypothesize that individuals living in a neighborhood with alcohol outlets will be at higher risk for both SA and SD and that the effects of proximity will be exacerbated among individuals at higher genetic liability to suicidal behavior. A substantial effect of outlet proximity on risk of suicidal behavior could have implications for alcohol-related policies.

## MATERIALS AND METHODS

### Sample

We collected longitudinal information on individuals from Swedish population-based registers with national coverage linking each person’s unique personal identification number which, to preserve confidentiality, was replaced with a pseudonymized number by Statistics Sweden. We secured ethical approval for this study from the Regional Ethical Review Board in Lund, and no participant consent was required as the analyses were based on nationwide secondary data (No. 2008/409 and later amendments). We created four different databases reflecting the information we have on alcohol outlets. The four databases included all individuals 18 to 25 years residing in Sweden at the end of 2005, 2008, 2010, and 2013, respectively.

### Measures

Data are derived from a range of nationwide Swedish registers, including the Multi-Generation Register, Longitudinal Integrated Database for Health Insurance and Labour Market Studies (LISA), Total Population Register, National Patient Register, and Primary Care Register. Further details are provided in the Supplementary Material.

### Primary predictor: neighborhood alcohol outlets

For all individuals, we included their neighborhood of residence at the end of 2005, 2008, 2010, and 2013. Neighborhoods were defined using Small Areas for Market Statistics (SAMS) obtained from Statistics Sweden, the Swedish government-owned statistics bureau. There are approximately 9400 SAMS throughout Sweden, with an average population of about 1000 inhabitants. The SAMS boundaries are drawn to include similar types of housing construction. For each SAMS, we have information on the presence/absence of three different types of alcohol outlets: bars, nightclubs, and government stores (similar to state-run alcohol beverage control stores in the United States; these outlets sell wine, liquor, and higher alcohol content beer than is available in grocery stores [3.5% alcohol by volume and higher]). Included in our definition of government store are local agents where individuals can preorder alcoholic beverages that are then delivered for pickup.

### Outcome: SA or suicide death

Suicide attempt was defined in the Swedish medical registers using the ICD-10 codes X60-X84 and Y10-Y34. We used the first date of registration of SA. We identified records of SD from the Swedish Mortality register, using the same ICD-10 codes as for SA. To be included as SA or SD in the database, the SA/SD had to occur within 2 years after baseline (i.e., for the 2005 database the SA/SD had to occur in 2006 or 2007). In the analyses of SA, we excluded all individuals that had a registration of SA prior to baseline, as our research question focused on whether exposure to alcohol outlets was associated with risk of first SA. Attempts that were followed by SD within 30 days were not counted as SA in the analyses to avoid mis-classifying attempts whose lethal impacts were delayed as nonfatal.

#### Covariates

We included biological sex at birth, year of birth, parental education, and neighborhood deprivation as covariates in each model. Parental education was calculated as the mean number of years of education across both parents of the proband and was standardized per year of birth to a mean of 0 and a standard deviation (SD) of 1. Neighborhood deprivation is a composite variable calculated at the level of each SAMS, reflecting the proportion of working-age residents in the SAMS with low education, proportion with low household income, proportion unemployed, and proportion receiving government financial assistance. Further details are provided in the Supplementary Material.

In addition, we included as a covariate a Family Genetic Risk Score (FGRS) for suicide attempt (FGRS_SA_) or suicide death (FGRS_SD_), depending on the outcome variable. FGRS reflect aggregate genetic risk and are derived by first identifying first- to fifth-degree relatives of the proband and determining their status on the variable of interest (here, SA or SD). We then conduct a series of corrections and transformations, for example to adjust for cohabitation and thereby reduce the potential impact of shared environment on familial resemblance. Each step in this process is described in detail in the Supplementary Material. Ultimately, scores were standardized by year of birth and county of proband residence into a *z*-score with a mean of 0 and SD of 1; these standardized scores were included in our models.

### Statistical approach

To estimate the influence of alcohol outlets on future SA/SD, we used logistic regression models. In the models, we included three dummy variables for the presence/absence of bars, nightclubs, and government stores. Additionally, we controlled for year of birth, sex, neighborhood deprivation, parental education, and FGRS_SA_/FGRS_SD_ as noted above. To combine results from the four different samples, we used a standard meta-analytical approach, implemented using the *meta* package in R (Schwarzer et al., 2015). We calculated the combined odds ratio (OR) and the *p*-values for the heterogeneity tests that evaluate the null hypothesis that effects are similar across samples. Where evidence of a consistent main effect of alcohol outlet was detected, we specified a follow-up model that includes an interaction term between the pertinent FGRS and the implicated alcohol outlet. In these models, we calculated the relative excess risk due to interaction (RERI). Analyses were conducted in SAS Version 9.4 and Rstudio Version 2022.02.2.

## RESULTS

### Descriptive statistics

Across the 4 years for which information on alcohol outlets were available (2005, 2008, 2010, and 2013), SA data were available for *N* = 347,938 to 420,115 females and *N* = 371,088 to 447,719 males (Table 1). The rate of first SA within each two-year observation period was relatively consistent across periods. Rates of SA were similar across the sexes, likely reflecting the fact that registry data primarily captures medically serious attempts. Corresponding data for SD are provided in Table 2: The sample size for females ranged from *N* = 355,929 to 433,441, and for males from *N* = 376,647 to 458,378. The rate of SD was consistent across observation periods and was higher among males relative to females.

While the number of bars and government alcohol outlets generally increased gradually across observation periods, the number of nightclubs varied widely. We could not identify a clear explanation for this fluctuation. We have retained this variable in our analyses, but findings should be considered with caution.

### Logistic regressions with SA as the outcome

We used logistic regressions to estimate the association between each alcohol outlet type and SA. Odds ratios (ORs) and 95% confidence intervals (CIs) are provided in Table 3 and Figure S1 for each of four 2-year observation periods, based on the availability of data on the presence/absence of each type of alcohol outlet at the beginning of that year (2005, 2008, 2010, or 2013), along with estimates pooled across observation periods. For the total sample, the point estimates for bars were consistently elevated but confidence intervals overlapped 1 in each observation period. The pooled estimate was significantly greater than 1 in the full sample (i.e., with females and males combined). Odds ratio estimates for nightclubs did not differ significantly from the null hypothesis in any individual observation period or for the pooled estimate. The OR for government outlets exceeded 1 in four cases: (i) for males in the observation period beginning in 2010; (ii) for the sexes combined during that same period; (iii) for males, pooled across observation periods; and (iv) in the sexes combined pooled across observation periods. We did not observe significant heterogeneity across observation periods.

Later birth years were associated with higher risk of SA without exception, with ORs slightly higher among females. In addition, higher parental education was consistently associated with lower risk of SA. Higher neighborhood deprivation was largely associated with increased SA risk, though this effect was not significant in the male-only analysis. The highest effect size was observed for FGRS_SA_, which was consistently associated with higher risk of SA.

Consistent with our analytic plan, we conducted follow-up analyses to evaluate whether individuals at higher genetic liability to SA (FGRS_SA_) were more sensitive to the effect of having an alcohol outlet in their neighborhood. We tested interaction terms for each of the five models in which we observed a main effect of an alcohol outlet, resulting in two significant interactions: (i) between government outlets and FGRS_SA_ in the sexes combined, pooled across observation periods (RERI = 0.05; 95% CI 0.01; 0.09); and (ii) between governmental outlets and FGRS_SA_ in males, pooled across observation periods (RERI = 0.06; 95% CI 0.001; 0.12). In both cases, individuals with higher FGRS_SA_ were more sensitive to the effects of living in a neighborhood with a government alcohol outlet. Results for all tests are provided in Table S1.

### Logistic regressions with SD as the outcome

We next conducted logistic regressions to estimate the association between neighborhood alcohol outlets and SD, with results presented in Table 4 and Figure S2. Within the total sample, ORs for each outlet type were less consistent across observation periods than observed in models with SA as the outcome, potentially due to the small number of suicide cases. As with SA, we did not observe significant associations between nightclubs and SD. In contrast to SA, the pooled estimates for bars among the sexes combined and the male subsample were *inversely* associated with risk of SD. Consistent with the SA models, we observed a significant positive association between governmental alcohol outlets and SD during the observation period beginning in 2010 for the sexes combined and for males. The effect size was stronger for SD (see Table 4).

In contrast to findings for SA, the effect of later year of birth varied in models with SD as the outcome. In the total sample, ORs for three of the four observation periods were <1, as was the overall OR (OR = 0.96, 95% CI 0.93; 0.98), such that later birth year was associated with *lower* risk of SD. Sex-stratified results suggest that this association was driven by the male subsample. Higher parental education was overall associated with lower risk of SD in the total sample, though in each observation period, and within sex-stratified analyses, confidence intervals included 1 in most cases. As observed for the SA models, higher FGRS_SD_ was consistently associated with higher risk of SD.

Where we observed main effects of alcohol outlets, we tested corresponding interaction terms with FRGS_SD_. We observed limited significant interactions (see Table S1).

## DISCUSSION

In the current study, we aimed to expand upon prior research exploring the effects of proximity to alcohol outlets on suicidal behavior in several key ways: (i) examining individual-level risk, rather than associations observed at the aggregate level; (ii) expanding to include nonfatal SA as an outcome in addition to SD; (iii) jointly testing the effects of multiple types of alcohol outlets; and (iv) conducting sex-stratified analyses. Our findings provide only modest support for the hypothesis that residing in close proximity to an alcohol outlet as a young adult increases risk of suicidal behavior in the subsequent 2 years. Specifically, across four observation periods, we found evidence that bars are associated with increased risk of nonfatal attempt when female and male samples were combined, but with decreased risk of SD—a result driven by males. Furthermore, governmental alcohol outlets were associated with increased risk of SA and SD for one observation period, and when pooling across periods. In sex-stratified analyses, this effect was limited to males, though the direction of effect was consistent across sexes. Thus, while other sociodemographic features were associated with SA and/or SD risk, living in a neighborhood with an on- or off-premise alcohol outlet was associated with a minimal increase in one’s risk of suicidal behavior during young adulthood. In some cases, the effect of living near an alcohol outlet was slightly exacerbated among those with higher genetic liability to SA.

The current findings are consistent with at least four prior studies in that the effects of alcohol access on suicide risk were limited to or driven by males. Markowitz et al. (2003) reported that male youth suicides in the United States were lower under conditions of higher excise taxes on alcohol and a zero-tolerance approach to drunk driving. In addition, Pridemore and colleagues reported suicide reductions in males only, corresponding to alcohol accessibility in Slovenia (Pridemore & Snowden, 2009) and to alcohol pricing in Russia (Pridemore et al., 2013). Finally, using data from 14 states participating in the National Violent Death Reporting System, Giesbrecht et al. (2015) reported positive associations between off-premise alcohol outlet density and the presence of alcohol among male, but not female, suicide decedents. Notably, our analyses included all SDs among the cohort, not only those where alcohol was detected in the decedent. The absence of an effect among females in the current and some prior studies could be due to lack of statistical power, as SDs are more common among males than females (National Institute of Mental Health, 2022); in the current study, the direction of effect was mostly–but not always–consistent across sexes, with confidence intervals generally wider among females for estimates relating to SD.

The small effects we observed were limited to bars, which were associated with increased risk of SA and decreased risk of SD; and to government alcohol outlets, which were associated with increased risk of both SA and SD. The latter sell liquor, wine, and beer, including beer with greater than 3.5% ABV, which in Sweden is not sold in other retail outlets such as grocery stores. Individuals must be at least 20 years of age to purchase alcohol from government outlets, whereas bars and nightclubs are able to sell alcohol to those aged 18 and older. Government outlets’ operating hours are also more limited than other outlets. This suggests that policy changes that impact a range of outlet types may be effective in reducing suicidal behavior–for example, tax increases on alcohol rather than a reduction in hours for government outlets. However, we caution against overinterpretation of the current findings, and such efforts are likely not warranted on the basis of the current results, given the small effect sizes and inconsistency of effects across observation periods.

One notable and unexpected result was the association between proximity to bars and *decreased* risk of SD, an effect that was driven by males. These effects were only detectable in pooled estimates, underscoring the low magnitude and relative imprecision of the effect. While inconsistent with much of the prior literature, such an inverse association has been found previously. A Swedish study reported that, although alcohol consumption increased from 1994 to 2002 as alcohol became more accessible, this corresponded to a modest decrease in SDs (Andreasson et al., 2006). In addition, studies of Alaska Natives (Berman et al., 2000) and Alabama residents (Joubert, 1994) found that more permissive alcohol laws were associated with lower suicide rates. However, the opposite direction of effect for SA versus SD is difficult to explain. Prior research indicates that environmental factors contributing to SA and SD are only modestly correlated (rE = 0.21 to 0.36) (Edwards et al., 2021); the current findings suggest that alcohol accessibility might be one factor that differentially impacts SA versus SD risk, though the specific aspects of accessibility that might be relevant–for example, hours of operation and catering to a younger clientele–cannot be determined using the current data. Additional research is necessary to understand the range of factors contributing to this result.

What might explain the discrepancy between the current, tempered results and prior studies that found more robust evidence that increased alcohol accessibility can lead to higher rates of suicidal behavior? First, we focused on young adults, reasoning that this age group is more likely to consume alcohol in bars and nightclubs relative to older individuals, and we should thus have more discriminatory power for those outlets. Prior positive findings could be driven by older adults, who might disproportionately turn to government outlets for alcohol acquisition. Second, our exposure variables were binary, while several earlier efforts with positive results included as the exposure variable the number or density of alcohol outlets (Markowitz et al., 2003; Wasserman et al., 1994, 1998)–a more refined measure with greater statistical power. It is possible that localities with especially high numbers of alcohol outlets drive increased suicide rates, while the availability of only one or a few outlets has no appreciable impact on risk. Third, there may be confounding factors present at the aggregate level that are jointly associated with the presence of neighborhood alcohol outlets and higher risk of suicidal behavior, which have not been adequately accounted for previously. For example, in the current study, individuals living in high deprivation neighborhoods were generally (though not always) at increased risk of suicidal behavior, while individual socioeconomic position was inversely associated with SA; such variables have frequently been excluded in prior studies, leading to potential bias (Kolves et al., 2020). Furthermore, we were able to account for aggregate genetic liability to SA or SD; this variable consistently had one of the largest effect sizes of any variable in the model, and we are unaware of its inclusion in any prior study examining the association between alcohol access and risk of suicidal behavior.

Our findings should be considered in light of several limitations. First, information on alcohol outlets was only available for the years included in these analyses (i.e., 2005 and 2008), precluding our ability to determine with precision whether a neighborhood went from having no outlets to having one or more, or vice versa, within the observation period. We made an effort to mitigate this by limiting the follow-up period to within 2 years of when an individual’s proximity to various alcohol outlets was recorded: A longer follow-up would have expanded the time frame during which the status could change. Second, we were unable to determine the precise number of alcohol outlets in a neighborhood, leading to our use of a binary variable to indicate whether a particular neighborhood had *no* outlets versus *any* outlets at the designated time point. As some prior studies have reported the number or density of outlets is related to risk, further exploration using a measure with greater specificity is warranted. Third, drinking cultures vary across countries; the current results might not generalize to other samples, though they do reflect associations observed among a large cohort of young Swedish adults. Finally, despite our considerable sample size, suicidal behavior–in particular, SD–is a relatively uncommon event, which resulted in imprecise parameter estimates. Furthermore, had we relied on P-values rather than confidence intervals to determine effects of interest, some reported effects might not survive correction for multiple tests. We recommend interpreting these findings with a degree of caution, as replication is necessary in other samples.

In summary, we observed weak but significant evidence that proximity to specific types of alcohol outlets is associated with small increases in risk of nonfatal SA (bars and government outlets) and SD (government outlets) among young Swedish adults. However, living in a neighborhood with bars was also weakly associated with decreased risk of SD. All significant findings were driven by males. Importantly, effect sizes were low and, in some cases, inconsistent across observation periods, underscoring the need for additional research. Future studies should consider whether similar effects are observed in older individuals and whether the number or density of outlets is additionally informative for risk.

## Supplementary Material

Supplement 1

## Figures and Tables

**TABLE 1:** Descriptive statistics for sample included in logistic regressions with suicide attempt as the outcome.

	Year of proximity to alcohol outlet assessment			
	2005	2008	2010	2013
Total sample				
*N*	719,026	789,054	843,360	867,834
Suicide attempt within 2 years	0.6%	0.6%	0.6%	0.8%
Year of birth	1984 (2.3)	1987 (2.3)	1989 (2.3)	1991 (2.2)
*N* with bar in neighborhood	86,269 (12.0%)	100,474 (12.7%)	119,969 (14.2%)	123,879 (14.3%)
*N* with nightclub in neighborhood	5397 (0.8%)	30,556 (3.9%)	39,885 (4.7%)	15,381 (1.8%)
*N* with governmental alcohol outlet in neighborhood	68,883 (9.6%)	71,737 (9.1%)	78,875 (9.4%)	73,219 (8.4%)
FGRS_SA_(SD)	−0.01 (0.9)	−0.01 (0.9)	−0.01 (0.9)	−0.00 (0.9)
Neighborhood deprivation (SD)	0.13 (1.4)	0.14 (1.5)	0.14 (1.6)	0.12 (1.6)
Parental education (SD)	0.01 (0.08)	−0.02 (0.8)	−0.03 80.8)	−0.04 (0.8)

Females				
*N*	347,938	381,321	407,649	420,115
Suicide attempt within 2 years	0.7%	0.6%	0.6%	0.7%
Year of birth	1984 (2.3)	1987 (2.3)	1989 (2.7)	1991 (2.2)
*N* with bar in neighborhood	43,450 (12.5%)	50,130 (13.2%)	59,991 (14.7%)	61,994 (14.8%)
*N* with nightclub in neighborhood	2766 (0.8%)	15,570 (4.1%)	20,163 (5.0%)	7831 (1.9%)
*N* with governmental alcohol outlet in neighborhood	33,566 (9.7%)	34,720 (9.1%)	38,271 (9.4%)	35,679 (8.5%)
FGRS_SA_ (1 SD)	−0.01 (0.9)	−0.01 (0.9)	−0.01 (0.9)	−0.01 (0.9)
Neighborhood deprivation (SD)	0.14 (1.4)	0.14 (1.6)	0.15 (1.6)	0.13 (1.6)
Parental education	0.01 (0.8)	−0.02 (0.8)	−0.03 (0.8)	−0.04 (0.8)

Males				
*N*	371,088	407,733	435,711	447,719
Suicide attempt within 2 years	0.6%	0.6%	0.6%	0.8%
Year of birth	1974 (2.3)	1987 (2.3)	1989 (2.3)	1991 (2.2)
*N* with bar in neighborhood	42,819 (11.5%)	50,344 (12.4%)	59,978 (13.8%)	61,885 (13.8%)
*N* with nightclub in neighborhood	2631 (0.7%)	14,986 (3.7%)	19,722 (4.5%)	7550 (1.7%)
*N* with governmental alcohol outlet in neighborhood	35,317 (9.5%)	37,017 (9.1%)	40,604 (9.3%)	37,540 (8.4%)
FGRS_SA_ (SD)	−0.01 (0.9)	−0.01 (0.9)	−0.01 (0.9)	−0.00 (0.9)
Neighborhood deprivation (SD)	0.12 (1.4)	0.13 (1.5)	0.13 (1.6)	0.12 (1.6)
Parental education (SD)	0.01 (0.9)	−0.01 (0.8)	−0.03 (0.8)	−0.03 (0.8)

*Note:* Individuals were observed for 2 years beginning with the year after which proximity to alcohol outlets was measured.

Abbreviations: FGRS_SA_, family genetic risk score for suicide attempt; SD, standard deviation.

**TABLE 2:** Descriptive statistics for sample included in logistic regressions with suicide death as the outcome.

	Year of proximity to alcohol outlet assessment			
	2005	2008	2010	2013
Total sample				
*N*	732,596	808,007	865,182	891,819
Suicide death within 2 years	0.03%	0.03%	0.03%	0.03%
Year of birth	1984 (2.3)	1987 (2.3)	1989 (2.3)	1991 (2.2)
*N* with bar in neighborhood	88,126 (12.0%)	103,164 (12.8%)	123,525 (14.3%)	127,777 (14.3%)
*N* with nightclub in neighborhood	5481 (0.8%)	31,399 (3.9%)	41,040 (4.7%)	15,973 (1.8%)
*N* with governmental alcohol outlet in neighborhood	70,381 (9.6%)	73,699 (9.1%)	81,275 (9.4%)	75,569 (8.5%)
FGRS_SD_ (SD)	0.01 (1.0)	0.01 (1.0)	0.01 (1.0)	0.01 (1.0)
Neighborhood deprivation (SD)	0.13 (1.4)	0.15 (1.6)	0.15 (1.6)	0.13 (1.6)
Parental education (SD)	0.01 (0.8)	−0.02 (0.8)	−0.03 (0.8)	−0.04 (0.8)

Females				
*N*	355,949	392,371	420,192	433,441
Suicide death within 2 years	0.02%	0.02%	0.02%	0.02%
Year of birth	1984 (2.3)	1987 (2.3)	1989 (2.3)	1991 (2.2)
*N* with bar in neighborhood	44,511 (12.5%)	51,663 (13.2%)	61,978 (14.8%)	64,164 (14.8%)
*N* with nightclub in neighborhood	2816 (0.8%)	16,065 (4.1%)	20,831 (5.0%)	8136 (1.9%)
*N* with governmental alcohol outlet in neighborhood	34,425 (9.7%)	35,847 (9.1%)	39,625 (9.4%)	36,957 (8.5%)
FGRS_SD_ (1 SD)	0.01 (1.0)	0.01 (1.0)	0.01 (1.0)	0.01 (1.0)
Neighborhood deprivation (SD)	0.14 (1.4)	0.16 (1.6)	0.16 (1.6)	0.14 (1.6)
Parental education	0.00 (0.8)	−0.02 (0.8)	−0.03 (0.8)	−0.04 (0.8)

Males				
*N*	376,647	415,636	444,990	458,378
Suicide death within 2 years	0.05%	0.05%	0.05%	0.05%
Year of birth	1984 (2.3)	1987 (2.3)	1989 (2.3)	1991 (2.2)
*N* with bar in neighborhood	43,615 (11.6%)	51,501 (12.4%)	61,547 (13.8%)	63,613 (13.9%)
*N* with nightclub in neighborhood	2665 (0.7%)	15,334 (3.7%)	20,209 (4.5%)	7837 (1.7%)
*N* with governmental alcohol outlet in neighborhood	35,956 (9.6%)	37,852 (9.1%)	41,650 (9.4%)	38,612 (8.4%)
FGRS_SD_ (SD)	0.01 (1.0)	0.01 (1.0)	0.01 (1.0)	0.01 (1.0)
Neighborhood deprivation (SD)	0.12 (1.4)	0.13 (1.5)	0.14 (1.6)	0.12 (1.6)
Parental education (SD)	0.01 (0.8)	−0.02 (0.8)	−0.03 (0.8)	−0.04 (0.8)

*Note*: Individuals were observed for 2 years beginning with the year after which proximity to alcohol outlets was measured.

Abbreviations: FGRS_SA_, family genetic risk score for suicide attempt; SD, standard deviation.

**TABLE 3:** Results from logistic regressions with first suicide attempt as the outcome.

	Year of proximity to alcohol outlet assessment				Overall OR	Test of heterogeneity
	2005	2008	2010	2013		
Total sample						
Year of birth	1.09 (1.08; 1.11)	1.08 (1.07; 1.09)	1.07 (1.06; 1.08)	1.06 (1.05; 1.07)	1.07 (1.07; 1.08)	0.008
Bars	1.10 (1.00; 1.21)	1.02 (0.93; 1.13)	1.07 (0.98; 1.17)	1.03 (0.95; 1.11)	1.05 (1.01; 1.10)	0.633
Nightclubs	1.05 (0.74; 1.47)	0.98 (0.83; 1.15)	0.89 (0.77; 1.03)	0.96 (0.79; 1.16)	0.94 (0.86; 1.03)	0.744
Governmental outlet	1.08 (0.98; 1.20)	1.06 (0.95; 1.17)	1.13 (1.02; 1.24)	1.07 (0.98; 1.17)	1.08 (1.03; 1.14)	0.807
FGRS_SA_ (1 SD)	1.38 (1.35; 1.41)	1.38 (1.36; 1.41)	1.32 (1.29; 1.34)	1.22 (1.20; 1.25)	1.32 (1.31; 1.34)	<0.0001
Neighborhood deprivation (SD)	1.04 (1.03; 1.06)	1.04 (1.03; 1.06)	1.03 (1.01; 1.05)	0.99 (0.97; 1.00)	1.02 (1.01; 1.03)	<0.0001
Parental education	0.83 (0.80; 0.86)	0.83 (0.80; 0.86)	0.83 (0.80; 0.86)	0.81 (0.78; 0.84)	0.83 (0.81; 0.84)	0.651
Males versus females	0.92 (0.87; 0.98)	1.02 (0.96; 1.07)	1.02 (0.96; 1.07)	1.22 (1.16; 1.28)	1.05 (1.03; 1.08)	<0.0001

Females						
Year of birth	1.14 (1.12; 1.17)	1.12 (1.10; 1.14)	1.10 (1.08; 1.12)	1.09 (1.07; 1.10)	1.11 (1.10; 1.12)	0.002
Bars	1.10 (0.97; 1.26)	0.98 (0.86; 1.13)	1.08 (0.95; 1.22)	1.04 (0.93; 1.17)	1.05 (0.99; 1.12)	0.645
Nightclubs	1.08 (0.68; 1.72)	1.12 (0.89; 1.41)	0.80 (0.64; 1.00)	1.04 (0.79; 1.37)	0.97 (0.85; 1.11)	0.169
Governmental outlet	1.09 (0.94; 1.25)	0.97 (0.83; 1.13)	1.10 (0.95; 1.26)	1.08 (0.94; 1.24)	1.06 (0.99; 1.14)	0.614
FGRS_SA_ (1 SD)	1.33 (1.29; 1.37)	1.37 (1.33; 1.40)	1.31 (1.27; 1.34)	1.23 (1.20; 1.27)	1.31 (1.29; 1.33)	<0.0001
Neighborhood deprivation	1.07 (1.04; 1.10)	1.09 (1.06; 1.11)	1.05 (1.03; 1.07)	1.00 (0.98; 1.03)	1.05 (1.04; 1.06)	<0.0001
Parental education	0.85 (0.81; 0.89)	0.88 (0.83; 0.92)	0.86 (0.81; 0.90)	0.84 (0.80; 0.88)	0.86 (0.84; 0.88)	0.604

Males						
Year of birth	1.04 (1.02; 1.06)	1.05 (1.03; 1.06)	1.05 (1.03; 1.06)	1.04 (1.02; 1.05)	1.04 (1.04; 1.05)	0.729
Bars	1.09 (0.95; 1.25)	1.07 (0.94; 1.21)	1.07 (0.95; 1.20)	1.03 (0.93; 1.14)	1.06 (1.00; 1.13)	0.916
Nightclubs	1.01 (0.61; 1.67)	0.85 (0.68; 1.08)	0.99 (0.81; 1.20)	0.89 (0.68; 1.17)	0.93 (0.82; 1.05)	0.764
Governmental outlet	1.08 (0.93; 1.25)	1.14 (0.99; 1.31)	1.15 (1.01; 1.32)	1.06 (0.94; 1.20)	1.11 (1.03; 1.18)	0.814
FGRS_SA_ (1 SD)	1.43 (1.39; 1.47)	1.40 (1.36; 1.43)	1.32 (1.29; 1.36)	1.21 (1.18; 1.24)	1.34 (1.32; 1.35)	<0.0001
Neighborhood deprivation	1.02 (0.99; 1.05)	1.00 (0.98; 1.03)	1.01 (0.99; 1.04)	0.98 (0.96; 1.00)	1.00 (0.99; 1.01)	0.103
Parental education	0.81 (0.77; 0.85)	0.79 (0.75; 0.83)	0.81 (0.77; 0.85)	0.78 (0.75; 0.82)	0.80 (0.78; 0.81)	0.589

**TABLE 4:** Results from logistic regressions with SD as the outcome.

	Year of proximity to alcohol outlet assessment				Overall OR	Test of heterogeneity
	2005	2008	2010	2013		
Total						
Year of birth	1.00 (0.95; 1.06)	0.98 (0.93; 1.03)	0.93 (0.88; 0.97)	0.93 (0.88; 0.98)	0.96 (0.93; 0.98)	0.123
Bars	1.14 (0.77; 1.68)	0.86 (0.56; 1.33)	0.81 (0.55; 1.20)	0.89 (0.62; 1.27)	0.91 (0.89; 0.94)	<0.0001
Nightclubs	0.93 (0.22; 3.86)	1.27 (0.65; 2.49)	1.02 (0.56; 1.86)	0.94 (0.38; 2.34)	1.07 (0.73; 1.58)	0.943
Governmental outlet	1.29 (0.85; 1.94)	0.83 (0.51; 1.33)	1.47 (1.01; 2.14)	1.12 (0.74; 1.69)	1.19 (0.97; 1.47)	0.307
FGRS_SD_ (1 SD)	1.18 (1.11; 1.25)	1.14 (1.08; 1.21)	1.16 (1.10; 1.21)	1.13 (1.07; 1.19)	1.15 (1.12; 1.18)	0.712
Neighborhood SES	1.15 (1.07; 1.24)	1.07 (0.99; 1.14)	1.17 (1.11; 1.25)	1.10 (1.04; 1.17)	1.12 (1.09; 1.16)	0.216
Parental education	0.93 (0.80; 1.09)	0.83 (0.72; 0.97)	0.92 (0.80; 1.06)	0.98 (0.86; 1.13)	0.92 (0.85; 0.99)	0.448
Male versus female	2.19 (1.67; 2.87)	2.70 (2.06; 3.53)	2.83 (2.17; 3.69)	2.81 (2.18; 3.62)	2.63 (2.30; 3.00)	0.5041

Females						
Year of birth	1.07 (0.97; 1.19)	1.00 (0.90; 1.10)	1.03 (0.93; 1.14)	0.99 (0.89; 1.09)	1.02 (0.97; 1.07)	0.704
Bars	1.49 (0.77; 2.88)	0.81 (0.34, 1.92)	1.08 (0.53; 2.22)	0.82 (0.41; 1.64)	1.02 (0.97; 1.07)	<0.0001
Nightclubs	1.37 (0.18; 10.6)	0.87 (0.19; 4.01)	0.81 (0.23, 2.87)	1.32 (0.31; 5.69)	1.01 (0.47; 2.14)	0.947
Governmental outlet	1.10 (0.51; 2.37)	0.82 (0.31; 2.16)	1.18 (0.53; 2.61)	1.95 (0.99; 3.84)	1.29 (0.87; 1.91)	0.477
FGRS_SD_ (1 SD)	1.22 (1.12; 1.33)	1.10 (0.94; 1.28)	1.16 (1.08; 1.26)	1.13 (1.02; 1.25)	1.17 (1.11; 1.22)	0.563
Neighborhood deprivation	1.21 (1.06; 1.37)	1.06 (0.93, 1.22)	1.22 (1.09; 1.36)	1.10 (0.98; 1.24)	1.15 (1.08; 1.22)	<0.0001
Parental education	1.13 (0.86, 1.47)	0.87 (0.65; 1.16)	0.98 (0.74; 1.30)	0.72 (0.54; 0.97)	0.92 (0.80; 1.06)	0.149

Males						
Year of birth	0.97 (0.91; 1.04)	0.97 (0.91; 1.03)	0.89 (0.84; 0.95)	0.91 (0.86; 0.97)	0.93 (0.90; 0.96)	0.106
Bars	1.02 (0.62; 1.66)	0.90 (0.54; 1.48)	0.74 (0.47; 1.18)	0.94 (0.62; 1.43)	0.89 (0.86; 0.92)	<0.0001
Nightclubs	0.73 (0.10; 5.37)	1.45 (0.68; 3.07)	1.13 (0.57; 2.23)	0.81 (0.25; 2.61)	1.15 (0.73; 1.81)	0.822
Governmental outlet	1.37 (0.84; 2.23)	0.82 (0.47, 1.42)	1.56 (1.01; 2.39)	0.85 (0.50; 1.43)	1.16 (0.91; 1.49)	0.163
FGRS_SD_ (1 SD)	1.15 (1.06; 1.24)	1.16 (1.09; 1.24)	1.15 (1.01; 2.39)	1.13 (1.06; 1.20)	1.15 (1.1; 1.19)	0.954
Neighborhood deprivation	1.13 (1.03; 1.24)	1.07 (0.99; 1.16)	1.16 (1.08; 1.25)	1.11 (1.03; 1.19)	1.12 (1.08; 1.16)	<0.0001
Parental education	0.86 (0.71; 1.03)	0.82 (0.69; 0.98)	0.90 (0.76; 1.06)	1.08 (0.93; 1.26)	0.92 (0.85; 1.00)	0.096
