# Supplementary material for: Exposure to alcohol outlets and risk of suicidal behavior in a Swedish cohort of young adults
Source: Alcohol Clin Exp Res (Hoboken). Author manuscript; Available in PMC 2024 Mar 6. (PMC10916709; doi:10.1111/acer.15051)
Supplement: Supplement 1 [file NIHMS1963864-supplement-Supplement_1.docx]

**Supplementary Material**

**Supplemental Table.** Results from tests of interactions terms.

| Outcome | Exposure | Observation Period | Sex | RERI |
| --- | --- | --- | --- | --- |
| SA | Governmental outlets | 2010 | Combined | 0.07 (-0.01; 0.15) |
| SA | Bars | Pooled | Combined | 0.003 (-0.04; 0.05) |
| SA | Governmental outlets | Pooled | Combined | 0.05 (0.01; 0.09) |
| SA | Governmental outlets | 2010 | Males | 0.08 (-0.03; 0.19) |
| SA | Governmental outlets | Pooled | Males | 0.06 (0.001; 0.12) |
| SD | Governmental outlets | 2010 | Combined | 0.06 (-0.24; 0.36) |
| SD | Bars | Pooled | Combined | 0.00 (-0.07; 0.07) |
| SD | Governmental outlets | 2010 | Males | 0.05 (-0.34; 0.44) |
| SD | Bars | Pooled | Males | 0.00 (-0.10; 0.10) |

SA=suicide attempt; SD=suicide death; RERI=Relative Excess Risk due to Interaction

**Supplemental Figure 1.** Odds ratios and 95% confidence intervals for each model tested, where suicide attempt is the outcome. The black dashed line represents OR=1 (the null hypothesis). The y-axis is on the logarithmic scale.

**Supplemental Figure 2.** Odds ratios and 95% confidence intervals for each model tested, where suicide death is the outcome. The black dashed line represents OR=1 (the null hypothesis). The y-axis is on the logarithmic scale.
